# Telenutrition promoting equity of access in the SUS: a descriptive study

**DOI:** 10.1590/1516-3180.2025.3462.26012026

**Published:** 2026-04-10

**Authors:** Mariana Setanni Grecco, Laís Fileti Fraga, Mônica Rossatti Molina, Marcus Vinicius Dutra Zuanazzi, Camilla do Rosario Nicolino Chiorino, Soraya Camargo Ito Süffert

**Affiliations:** I Nutritionist, Proadi-SUS; BP – A Beneficência Portuguesa de São Paulo, São Paulo (SP), Brazil.; II Nutritionist, Proadi-SUS; BP – A Beneficência Portuguesa de São Paulo, São Paulo (SP), Brazil.; III Physician; Medical Coordinator, Proadi-SUS; BP – A Beneficência Portuguesa de São Paulo, São Paulo (SP), Brazil.; IV Physician; Medical Consultant, TeleNordeste; Proadi-SUS; BP – A Beneficência Portuguesa de São Paulo, São Paulo (SP), Brazil.; V Nurse; Project Manager, Proadi-SUS; BP – A Beneficência Portuguesa de São Paulo, São Paulo (SP), Brazil.; VI Physician; Medical Researcher, TeleNordeste; Proadi-SUS; BP – A Beneficência Portuguesa de São Paulo, São Paulo (SP), Brazil.

**Keywords:** Telenutrition, Referral and consultation, Effective access to health services, Digital public health, Public health, Primary care, Equity of access, Nutritional care, Sistema Único de Saúde, Proadi-SUS

## Abstract

**BACKGROUND:**

In the area of nutrition, telehealth regulations were established on October 22, 2023, through Resolution No. 760, which defines and regulates telenutrition as a form of care and/or provision of services in food and nutrition through Information and Communication Technologies.

**OBJECTIVES:**

To present the profile of telenutrition users in the TeleNordeste-BP project. Design and setting: Descriptive study carried out by hospital BP – A Beneficência Portuguesa de São Paulo.

**METHODS:**

All services were provided through teleconsultation with nutritionists from the TeleNordeste project between May and December 2023.

**RESULTS:**

Of the 884 services, 873 (98.8%) were tele-interconsultation. The distribution of services by state was: 430 (48.6%) from Maranhão; 301 (34%) from Alagoas and 153 (17.3%) are from patients from Piauí. Among the services provided, 671 (75.9%) users were female and 213 (24.1%) were male; the average age was 39.54 years (standard deviation +/− 19.9). Most adult patients were overweight: 135 (25.9%) were classified as overweight, 121 (23.2%) as grade 1 obese, 100 (19.2%) as grade 2 obese, and 66 (12.6%) as grade 3 obese.

**CONCLUSION:**

chronic non-communicable diseases (NCDs) were the main reasons for seeking nutritional services, with more than 90% of the main reasons for consultation. Access to the telenutrition service offered by the TeleNordeste-BP project not only impacted the patient in ensuring access to nutritional care but also enabled the increase of Vigilância Alimentar e Nutricional (VAN).

## INTRODUCTION

 The evolution of information systems and communication technologies has fostered healthcare development and addressed a major challenge in the provision of accessible, affordable, and high-quality healthcare services. Geographical barriers are no longer a limiting factor in accessing new resources and technologies.^1-3^ In the field of nutrition, telehealth showed some movements in 2009; however, it was from the COVID-19 pandemic that the discussion about its regulation took shape,^4^ and on October 22, 2023, the Conselho Federal de Nutrição published Resolution No. 760 defined and regulated telenutrition as a form of care and/or provision of services in food and nutrition through Information and Communication Technologies.^5^

 Considering the technological resources and expanded access to healthcare enabled by telehealth, Hospital BP – A Beneficência Portuguesa de São Paulo, in partnership with the Brazilian Ministry of Health through the Programa de Apoio ao Desenvolvimento Institucional do Sistema Único de Saúde (Proadi-SUS), implemented the TeleNordeste Project,^6^ in the states of Alagoas, Maranhão, and Piauí over a three-year period from 2021 to 2023. The project provided synchronous teleinterconsultations across multiple specialties to support Primary Health Care (PHC) services in 360 participating municipalities. 

 The inclusion of nutritionists in the TeleNordeste Project was designed to support the healthcare model and work processes through integration with Healthcare Networks, enabling the dietary monitoring of patients. Telenutrition facilitates access to nutritional therapy, while teleconsultations shared with other healthcare professionals promote a model of continuing education, including professional support, training, and coordinated care for users of the Unified Health System (SUS). 

 Within the project, the role of telenutrition was guided by the principles of the Política Nacional de Alimentação e Nutrição (PNAN), encompassing the organization of nutritional care; promotion of adequate and healthy eating; food and nutrition surveillance; participation and social control; and research, innovation, and knowledge production in food and nutrition, while respecting the essential and derived attributes of PHC.^7^

 In this context, the present descriptive study aims to characterize the profile of users assisted by nutrition services within the TeleNordeste-BP project, considering the reasons for care, nutritional status by age group, clinical outcomes, and user satisfaction. 

## METHODS

 This study adhered to the Strengthening of the Reporting of Observational Studies in Epidemiology (STROBE) Statement.^8^

### Study design

 Descriptive study analyzing variables collected during nutrition teleinterconsultations within the TeleNordeste Project. 

### Local

 This research was developed by Hospital BP – A Beneficência Portuguesa de São Paulo through the Programa de Apoio ao Desenvolvimento Institucional do Sistema Único de Saúde (Proadi-SUS), in partnership with the Brazilian Ministry of Health, as part of the implementation of the Specialized Medical Assistance Project in the Northeast region of Brazil via telemedicine (TeleNordeste-BP). The project was registered under NUP 25000.170151/2021-65 and operated in three states in the Brazilian Northeast: Alagoas, Maranhão, and Piauí. 

### Study period

 Data collection was conducted from May to December 2023. 

### Inclusion and exclusion criteria

 All nutrition teleinterconsultations conducted by the TeleNordeste Project during the study period were included. 

### Variables

 Variables were recorded during consultations and extracted from patients’ electronic medical records for subsequent analysis. The variables considered included consultation outcomes (resolution indicators, communication flow, and coordination with points of the Health Care Network); types of consultations provided (teleinterconsultations; triangulated consultations involving a primary care nutritionist, a primary care healthcare professional, and the patient; and teleconsultations for clinical case discussion between a nutritionist and a primary care healthcare professional); demographic and anthropometric data (sex, age, weight, and height); and project evaluation through satisfaction surveys using the Net Promoter Score (NPS)^9,10^ administered to users and healthcare professionals in the region. 

### Data analysis

 Statistical analyses were performed using PSPP-GNU^®^ statistical software (GNU General Public License, version 3, June 29, 2007). Continuous variables with a normal distribution were expressed as means and standard deviations, whereas continuous variables with a non-normal distribution were presented as medians and interquartile ranges. Categorical variables were reported as absolute frequencies and percentages. A 95% confidence interval (95% CI) considered significant when *p* < 0.05. 

### Research ethics committee approval

 The study protocol was reviewed and approved by the Research Ethics Committee of Hospital BP – A Beneficência Portuguesa de São Paulo (CAAE No. 72813923.6.0000.5483), with a waiver of informed consent. 

## RESULTS

 Data from 884 consultations were analyzed, of which 873 (98.8%) were conducted as teleinterconsultations and 11 (1.2%) were teleconsultations without the presence of the patient. The distribution of consultations by state was as follows: 430 (48.6%) from Maranhão, 301 (34%) from Alagoas, and 153 (17.3%) from Piauí. Among the consultations performed, 671 users (75.9%) were female and 213 (24.1%) were male, with a mean age of 39.54 years (standard deviation ± 19.9). Nutritional care was offered to users across all stages of life. Of the 884 patients treated, age data were missing for three patients (0.3%). The greatest demand was observed among adults, accounting for 577 consultations (65.3%), followed by elderly patients with 146 consultations (16.5%). Care for adolescents accounted for 83 users (9.4%), children for 56 (6.3%), and infants for 19 users (2.1%) (**Table 1**). 

**Table 1:** Characterization of the study population

**Total**		**884 (100%)**
Gender^*^		
	*Female*	671 (75.9%)
	*Male*	213 (24.1%)
	Age (years)^**^	39,54 (+/−19,9)
Age group^*^		
	*Infant*	19 (2.1%)
	*Child*	56 (6.3%)
	*Teenager*	83 (9.4%)
	*Adult*	577 (6.,3%)
	*Elderly*	146 (16.5%)
States^*^		
	*Alagoas*	301 (34%)
	*Maranhão*	430 (48.6%)
	*Piauí*	153 (17.3%)
Type of service^*^		
	*Teleconsultation*	11 (1.2%)
	*Teleinterconsultation*	873 (98.8%)
Main diagnoses^*^		
	*Systemic Arterial Hypertension (SAH)*	227 (25.7%)
	*Diabetes Mellitus (DM)*	265 (30%)
	*Dyslipidemia (DLP)*	204 (23.1%)
	*SAH + DM*	77 (8.71%)
	*SAH + DM + DLP*	55 (6.22%)
Outcome of care^*^		
	*Follow-up in the PHC unit*	16 (1.8%)
	*Referral to another specialist*	20 (2.3%)
	*Return*	848 (95.9%)
NPS^*^		89
	*Total responses*	264 (100%)
	*Promoters*	242 (91.7%)
	*Neutrals*	15 (5.7%)
	*Detractors*	7 (2.7%)

* Data are presented in absolute numbers and percentages;

** means and standard deviations;

and (***) medians and interquartile ranges.

 Most patients treated had chronic non-communicable diseases. Of the total, 265 patients (30.0%) had diabetes mellitus, 227 (25.7%) had systemic arterial hypertension, and 204 (23.1%) had dyslipidemia. Seventy-seven patients (8.7%) had both diabetes and hypertension, while 55 (6.2%) presented with the three associated conditions: hypertension, diabetes, and dyslipidemia. Among the patients treated, 68 (7.7%) were pregnant. Regarding consultation outcomes, only 16 patients (1.8%) were discharged after the first consultation, while 848 users (95.9%) required follow-up consultations, and 20 patients (2.3%) were referred to another specialist or point within the healthcare network. With respect to user satisfaction, 264 responses were obtained for the Net Promoter Score (NPS), of which 241 (91.7%) were promoters, 15 (5.7%) were neutral, and 7 (2.7%) were detractors (**Table 1**). 

**Table 2** presents the nutritional status of users according to age group. Among children under 10 years of age, 6 (8.1%) were classified as underweight, 46 (62.2%) as normal weight, 6 (8.1%) as overweight, and 14 (18.9%) as obese; data were missing for 2 children (2.7%). In the 10–20 year age group, nearly half of the adolescents treated (35; 47.3%) were classified as obese, 12 (16.2%) as overweight, 16 (21.6%) as normal weight, and 7 as underweight. Among the 68 pregnant women assisted, 31 (45.6%) were classified as obese, 20 (29.4%) as overweight, 13 (19.1%) as eutrophic, and 4 (5.9%) as underweight. A total of 522 consultations were provided to adults, of which data for 19 users (3.6%) could not be analyzed. Most adult users were overweight or obese: 135 (25.9%) were classified as overweight, 121 (23.2%) as having grade 1 obesity, 100 (19.2%) as having grade 2 obesity, and 66 (12.6%) as having grade 3 obesity. 

**Table 2:** Nutritional status by age group

**Population**		**Children/teenagers (years)**		**Pregnant women**	**Adults**	**Elderly**
Age range		0–10	10–20	≥ 10	≥ 20	≥ 60
Weight (kg)		15.9 (17.65)^***^	68,32 (28.88)^**^	74.55 (24.35)^***^	78 (27.3)^***^	69.86 (15.82)^**^
Height (m)		1.03 (0.42)^***^	1.55 (0.15)^***^	1.57 (0.06)^***^	1.57 (0.12)^***^	1.55 (0.11)^***^
BMI		16.35 (5.85)^***^	27.37 (9.41)^**^	29.9 (9.6)^***^	31.4 (10.2)^***^	28.78 (2.66)^**^
N total^*^		74 (100%)	74 (100%)	68 (100%)	522 (100%)	146 (100%)
Nutritional status^*^						
	*Underweight*	6 (8.1%)	7 (9.5%)	4 (5.9%)	17 (3.3%)	12 (8.2%)
	*Eutrophic*	46 (62.2%)	16 (21.6%)	13 (19.1%)	64 (12.3%)	39 (26.7%)
	*Overweight*	6 (8.1%)	12 (16.2%)	20 (29.4%)	135 (25.9%)	85 (58.2%)
	*Obesity*	14 (18.9%)	35 (47.3%)	31 (45.6%)	N/A	N/A
	*ND*	2 (2.7%)	4 (5.4%)	0 (0%)	19 (3.6%)	10 (6.8%)
	*Obesity I*	N/A	N/A	N/A	121 (23.2%)	N/A
	*Obesity II*	N/A	N/A	N/A	100 (19.2%)	N/A
	*Obesity III*	N/A	N/A	N/A	66 (12.6%)	N/A

* Data are presented in absolute numbers and percentages;

** means and standard deviations;

(***) medians and interquartile ranges

ND, not defined; N/A, not applicable.

 Among the elderly population, 146 consultations were conducted, with 85 users (58.2%) classified as overweight, 39 (26.7%) as eutrophic, and 12 (8.2%) as underweight; data were missing for 10 users (6.8%) (**Table 2**). 

 In Alagoas, 88 municipalities (86.3%) had nutritionist services, with 245 nutritionists registered in Primary Health Care (PHC). TeleNordeste provided nutrition teleinterconsultations in 19 municipalities in the state, three of which (15.8%) did not have nutritionists. In Maranhão, 177 municipalities (81.6%) had nutritionists, with 406 professionals registered in PHC. TeleNordeste provided services in 39 municipalities, nine of which (23.1%) did not have nutritionists. In Piauí, 159 municipalities (70.9%) had nutritionists registered in the National Registry of Health Establishments (CNES), with 281 professionals working in PHC. TeleNordeste delivered nutritional services in 30 municipalities, seven of which (23.3%) did not have nutritionists (**Table 3**). 

**Table 3:** Distribution of coverage of nutritionist assistance in the territory

		**State**		
		**Alagoas**	**Maranhão**	**Piauí**
Estimated population (inhabitants IBGE)^*^		3,220,104	7,010,960	3,271,199
Number of Municipalities (IBGE)^*^		102 (100%)	217 (100%)	224 (100%)
Municipalities with Nutritionists^*^		88 (86.3%)	177 (81.6%)	159 (70.9%)
	*Nutritionists in Primary Health Care*	245 (100%)	406 (100%)	281 (100%)
	*Nutritionists in Specialized Health Care*	393 (100%)	611 (100%)	309 (100%)
Municipalities served by TeleNordeste^*^		19 (100%)	39 (100%)	30 (100%)
	*No nutritionist in the municipality*	3 (15.8%)	9 (23.1%)	7 (23.3%)
	*With a nutritionist in the municipality*	16 (84.2%)	30 (76.9%)	23 (76.6%)

* Data are presented in absolute numbers or percentages.

## DISCUSSION

 Chronic non-communicable diseases (NCDs) were the primary reasons for seeking nutrition services, accounting for more than 90% of the main reasons for consultation. This finding is consistent with national data indicating that NCDs affect approximately 52% of individuals aged 18 years and older, with hypertension, back problems, depression, and diabetes being the most prevalent conditions.^11^ Evidence from the literature further indicates that cardiovascular diseases, diabetes, chronic kidney disease, and certain cancers are among the 10 leading causes of premature death (ages 30–69), and that diet represents a key modifiable risk factor.^12^ Therefore, nutritional care focused on the promotion, prevention, and control of NCDs constitutes a timely and relevant intervention for the region. 

 Another important finding of this study was the higher demand for teleinterconsultations among women, who accounted for 671 consultations (75.9%), with 61.3% of adult users classified as overweight or obese. In recent years, obesity prevalence among adults has more than doubled, increasing from 12.2% to 26.8%, while the prevalence of overweight individuals rose from 43.3% to 61.7% in 2019, disproportionately affecting women.^13^ A cross-sectional study by Brum et al.,^14^ published in 2025 and based on Vigitel data from 2006 to 2021, reported that compared with 2006, the prevalence in 2021 increased by 152% for BMI ≥ 45, 120% for BMI ≥ 40, and 104% for BMI ≥ 35. In contrast, BMI ≥ 30 increased by 66%, underscoring the growing burden of severe obesity and its implications for healthcare systems due to increased care demands. 

 Regarding children and adolescents, our study found that among children under 10 years of age, 18.9% were obese, and among those aged 10–20 years, 47.3% were obese. The prevention and treatment of childhood obesity has been a public health problem with growing numbers worldwide, increasing the risk of developing NCDs at this stage of life and in adulthood. According to data from the Ministry of Health, in 2020, 5.4% of children under 2 years of age monitored in PHC were severely underweight, and 15.5% were overweight or obese. The early introduction of solid foods, the provision of ultra-processed foods to this population, and socioeconomic factors have influenced this prevalence. Among infants aged 6–24 months, 44% had received ultra-processed foods.^15^

 Among adolescents aged 15–17 years, the prevalence of overweight was 19.4%, corresponding to an estimated total of 1.8 million people, and was higher among female adolescents (22.9%) than among male adolescents (16%). Regarding the obesity indicator, the pattern was similar to that observed for overweight, with prevalence being higher among female adolescents (approximately 8%) than among male adolescents (5.4%).^11^ The objective of nutrition actions among children should not only focus on improvements in anthropometric indices but also on behavioral changes.^15^ Considering the increase in obesity in Brazil, health policies aimed at preventing and treating obesity are necessary, and the study carried out by Flores-Ortiz et al.,^16^ published in 2019, suggests that such policies are especially needed in the capitals of the North, Northeast, and Central-West regions, where the greatest increases in the prevalence of overweight and obesity have occurred in the country. 

 The telehealth nutritional care of the TeleNordeste-BP project was structured based on the premises of in-person care and the presence of a professional from the territory, which assisted in the collection of reliable patient data, mainly anthropometric data. This enabled an assessment of the food and nutritional profile of the local population and its determining factors, supporting PHC in one of the guidelines of the Política Nacional de Alimentação e Nutrição (PNAN) for structuring health and nutrition indicators that guide the formulation of public health policies and local nutritional care actions, through the continuous description and prediction of trends in the food and nutrition situation and its determining factors.^17^

 Another relevant aspect regarding the presence of nutritionists in PHC was highlighted in a review conducted by Casas Agustench et al.^18^ in 2020, which noted that incorporating nutritionists into primary care settings, or increasing their presence, would provide access to more qualified health professionals to carry out nutritional treatment, representing a more cost-effective intervention in terms of health expenditures. Considering that in 2018 the total costs of hypertension, diabetes, and obesity in the SUS reached R$ 3.45 billion, and that 72% of these costs were attributable to individuals aged 30–69 years and 56% to women, the estimates of costs associated with chronic diseases related to inadequate nutrition highlight the economic impact of these conditions on the SUS^12^ and the importance of multidisciplinary actions aimed at lifestyle modification and health care. 

 In our study, according to the NPS assessment, the majority of users (91.7%) were promoters, and the NPS score was 89, indicating an excellent result. This finding is consistent with data in the literature showing that most patients perceive improved access to care^19,20^ and support the continuation of telehealth consultations in the future due to benefits such as reduced travel, lower costs, and guaranteed access to qualified care.^20^

 Regarding adherence to telenutrition services, care was provided in 88 (16.2%) of the 543 municipalities across the states of Alagoas, Maranhão, and Piauí. Adherence of municipalities to the TeleNordeste-BP project, the composition of local healthcare teams, and difficulties related to connectivity or access to technological devices may have influenced the number of municipalities that participated. Another important point is that approximately one in four municipalities that used the nutrition service did not have nutritionists in Primary Health Care, and the TeleNordeste-BP project enabled access to nutritional care for many users in these locations. 

### Limitations

 The results are subject to limitations related to the individual datasets used and the information recorded in the medical records, as well as to biases inherent in a retrospective study design. The number of nutritionists by region was estimated based on data extracted from the CNES database in March 2025. 

## CONCLUSION

 Chronic non-communicable diseases (NCDs) were the main reasons for seeking telenutrition services. Considering the socioeconomic impact involved, offering telenutrition consultations to SUS users is a relevant strategy for health promotion and prevention. Access to the telenutrition service offered by the TeleNordeste-BP project not only impacted patients by ensuring access to nutritional care but also enabled increased Vigilância Alimentar e Nutricional (VAN) in the municipalities served, providing data and evidence to support broader actions and public policies, such as regulating food advertising to children, taxing ultra-processed foods, and implementing intersectoral actions aimed at reducing food insecurity. 

## Data Availability

Data supporting the findings of this study are available from the corresponding author, Soraya Camargo Ito Süffert, upon request.
